# Sphenopalatine Ganglion Block for Headache Treatment After an Incidental Durotomy: A Case Report

**DOI:** 10.5152/TJAR.2023.21634

**Published:** 2023-02-01

**Authors:** Ana Marques, Inês Morais, Vânia Costa, Hugo Romão

**Affiliations:** 1 Department of Anaesthesiology, Centro Hospitalar de Vila Nova de Gaia (CHVNG), Vila Nova de Gaia, Portugal; 2 Department of Neurosurgery, Centro Hospitalar de Vila Nova de Gaia (CHVNG), Vila Nova de Gaia, Portugal

**Keywords:** Block, sphenopalatine ganglion, headache, postdural puncture, neurosurgery, spine

## Abstract

Incidental durotomy is the most common intraoperative complication of spine surgeries. Our main goal is to report a case of a postoperative postdural puncture headache following an incidental durotomy successfully managed with a sphenopalatine ganglion block. A 75-year-old woman, American Society of Anesthesiologists physical status II, proposed for a lumbar interbody fusion. During surgery, an incidental durotomy with cerebrospinal fluid leak occurred, being repaired with muscle and DuraSeal® Dural Sealant System. In the recovery room, 1 hour after the end of the surgery, the patient developed a severe headache associated with nausea and photophobia. A bilateral transnasal sphenopalatine ganglion block with 0.75% ropivacaine was performed. Immediate pain relief was verified. The patient reported only mild headaches on the first postoperative day, feeling progressively better until discharge. The sphenopalatine ganglion block may be an effective alternative treatment for postdural puncture headache following an incidental durotomy during neurosurgeries. Sphenopalatine ganglion block may be a safe, low-risk alternative in the treatment of postdural puncture headache after an incidental durotomy that can be used in the immediate postoperative period to enable early recovery and return to day-to-day activities, which will hopefully lead to better surgical outcomes and patient satisfaction.

**Main Points**

- Pain in the immediate postoperative period leads to late recovery and late return to day-to-day activities, which will lead to worse surgical outcomes and poor patient satisfaction.
- Sphenopalatine ganglion block may be a safe, low-risk alternative in the treatment of postdural puncture headache after an incidental durotomy.
- We suggest that in cases of incidental durotomy, a sphenopalatine ganglion block can be performed at the end of the surgery even before the excruciating pain occurs.

## Introduction

Incidental durotomy (ID) is the most common intraoperative complication of spine surgeries. The incidence of ID ranges from 0.3% for lumbar discectomy to 11% in spinal deformity correction surgeries. Higher incidence of ID is associated with age, obesity, diabetes mellitus, surgical invasiveness, number of levels, revision surgeries, and less experienced surgeons. Incidental durotomy is associated with short- and long-term sequelae such as persistent cerebrospinal fluid (CSF) leaks, headaches, pseudomeningocele formation, dural cutaneous fistulas, meningitis, epidural abscess, and new-onset neurological deficits. It ultimately affects the surgical outcome and patients’ postoperative quality of life.^1^

According to the Monro–Kellie hypothesis, the sum of the volumes of the brain, CSF, and intracranial blood compartments is a constant value. If the volume of one of these reduces, the others must increase to maintain an equilibrium.^2^ After an ID, CSF’s volume decreases, and compensatory vasodilation is seen. This vasodilation is parasympathetically mediated by neurons in the sphenopalatine ganglion (SPG), and it is responsible for the headache felt after an ID. Traction on pain-sensitive intracranial structures also contributes to the pain. By attenuating the parasympathetic activity with an SPG block, the vasodilation is addressed, and the patient achieves symptomatic relief.^3^ Another possible effect is the modulation of sensory processes in the trigeminal nucleus caudalis via afferent sensory fibres, which is responsible for a change in pain modulation and reduced central sensitisation to pain.^4^

The standard postdural puncture headache (PDPH) treatment consists in adopting a supine position, hydration, analgesics, caffeine, sumatriptan, laxatives, and abdominal binders which are rarely effective and the epidural blood patch which is demanding, invasive, and often painful.^3,5^

Sphenopalatine ganglion block has emerged as a noninvasive alternative to this treatment. Our main goal is to report a case of a postoperative PDPH following an ID successfully managed with an SPG block.

## Case Presentation

After patient’s informed written consent, we report the case of a 75-year-old woman, American Society of Anesthesiologists (ASA) physical status II, with a past medical history of dyslipidemia and obesity (body mass index 30 kg m^-2^) and a previous surgical history of removal of a left L4–L5 disc herniation and posterior fixation.

She was now proposed for posterior lumbar interbody fusion of L3–L4 and L4–L5. Following the patient’s arrival at the operating room and confirmation of a functioning intravenous (IV) access, the patient was monitored according to ASA standards. A bilateral L3–L4 ultrasound-guided erector spinae plane block was performed with the patient in prone position. A total of 40 mL of 0.5% ropivacaine (100 mg) was injected deep into the erector spinae muscle. Following this, balanced general anaesthesia was performed, with no intercurrences.

The surgical technique consisted of the placement of pedicle screws in L3, L4, and L5 and removal of the L4 and L5 spinous apophysis as well as the pre-existing interlaminar stabilisation dispositive. An L3 and L4 laminectomy and flavectomy and an L3–L4 facetectomy were performed followed by decompression of the recesses and foramina, bilaterally from L3 and L4. During this step, an incidental anterior durotomy occurred. Due to suturing difficulties, the tear was repaired with muscle and DuraSeal® Dural Sealant System.

The laborious surgery lasted for 5 hours with an estimated blood loss of 1000 mL. In the intraoperative period, paracetamol 1000 mg IV, tramadol 100 mg IV, ketorolac 30 mg IV, and ketamine 0.25 mg kg^-1^ IV were administered. By the end of the surgery, the patient awoke with no complaints of low back pain or headache.

In the recovery room, approximately 1 hour after the end of the surgery, a new onset of a severe occipital headache (rated 9/10 on the Numeric Rating Scale) associated with nausea and photophobia was objectivated. There was no neck stiffness, dysphasia, low back pain, motor deficits, or any other relevant findings on clinical examination.

Since an ID occurred during the surgery, a PDPH was assumed and an SPG block was performed, guided by anatomical references, after discussion with the neurosurgeon. Life-threatening aetiologies like haemorrhage, thrombosis, vasculopathy, and meningitis were less probable since there were no focal neurologic deficits.

A transnasal approach with 0.75% ropivacaine, 2.5 mL in each nostril, was used. Two cotton tip applicators were placed in the nasopharynx’s posterior wall at the level of the middle turbinate for 12 minutes. The technique was well tolerated, causing no discomfort, oropharyngeal paresthesia, bleeding, or any other side effects. Pain relief was immediate, resulting in a Numeric Rating Scale of 3. The patient reported no nausea, vomiting, visual symptoms, or any other complications. New onset of neurological signs, changes in the headache nature, or conscious level fluctuations should prompt further investigation and imaging.

During the next 5 days, the patient received paracetamol 1000 mg IV every 8 hours, ketorolac 30 mg IV every 8 hours, tramadol 100 mg IV every 12 hours, and diazepam 5 mg IV once a day. She reported only mild headaches on the first postoperative day (Numeric Rating Scale of 3/10), feeling progressively better until discharge.

## Discussion

Severe PDPH is debilitating, preventing early mobilisation and rehabilitation and may mask signs of serious complications. Although conservative measures and epidural blood patch are presently considered the therapeutic gold standard,^6^ newer and less invasive treatments like the SPG block are gaining popularity.

The SPG is a parasympathetic ganglion, triangular-shaped, located in the pterygopalatine fossa, posterior to the middle nasal turbinate and anterior to the pterygoid canal. Postganglionic parasympathetic and sympathetic neurons and the somatic sensory afferents can be blocked by an SPG block.^3^

To perform an SPG block, a cotton swab soaked in ropivacaine or lidocaine is inserted parallel to the floor of the nose until resistance is encountered. At this point, the swab will be at the posterior pharyngeal wall superior to the middle turbinate. The applicator should be retained for 5-10 minutes.^3^ Sphenopalatine ganglion block can also be performed under direct endoscopic view for a more effective block. This technique consists of SPG bilateral direct injection with a local anaesthetic, via an endonasal endoscope, allowing the needle to directly penetrate the pterygopalatine fossa minimising extraneous absorption through the nasopharynx.^7^

Complications are usually rare and minor (bleed, transient discomfort, anaesthesia of the pharynx), and there are almost no contraindications (local infection, skull base fractures).^3^ The SPG block effective duration is approximately 8 hours, and it should be considered as a temporary measure to relieve symptoms until the dural puncture site generates sufficient granulation tissue to obstruct the outflow of CSF.^8^

The transnasal SPG block has been traditionally used to treat acute and chronic medical conditions, like migraine, cluster headache, trigeminal neuralgia, and atypical facial pain. It has also shown good results in recent case series to treat acute PDPH headache in obstetric patients and in other cases of neuraxial anaesthesia and diagnostic lumbar punctures.^3^ Recently, it proved to be an effective technique in reducing early post operative nausea and vomiting in endoscopic sinus surgery patients.^7^

However, there are no reports of the SPG block applied to ID as a neurosurgical complication. To our knowledge, there is only one report of an SPG block in a neurosurgical case: it reports successful use of an SPG block in a PDPH secondary to a lumboperitoneal shunt performed to treat intracranial hypertension due to a venous sinus thrombosis.^6^

In our case, a transnasal approach with a total of 5 mL of 0.75% ropivacaine applied for 12 minutes provided immediate and sustained pain relief without any complications. In addition, our patient presented various risk factors for ID like age, obesity, previous spine surgical history, and surgical invasiveness. We suggest that in cases like this if an ID occurs, the possibility of an SPG block at the end of the surgery should be discussed, even before the excruciating pain occurs.

In conclusion, our clinical case suggests that sphenopalatine ganglion block may be a safe, low-risk alternative in the treatment of PDPH after an ID that can be used in the immediate postoperative period to enable early recovery and return to day-to-day activities, which will hopefully lead to better surgical outcomes and patient satisfaction.

Future randomised, case-control studies are needed to ascertain SPG block efficacy in PDPH associated with neurosurgical complications.
